# Online counselling for family carers of people with young onset dementia: The RHAPSODY-Plus pilot study

**DOI:** 10.1177/20552076231161962

**Published:** 2023-03-08

**Authors:** Stephanie Perin, Rhoda Lai, Janine Diehl-Schmid, Emily You, Alexander Kurz, Maria Tensil, Michael Wenz, Bettina Foertsch, Nicola T Lautenschlager

**Affiliations:** 1 Academic Unit for Psychiatry of Old Age, Department of Psychiatry, 2281 The University of Melbourne, Melbourne, Australia; 2 Department of Psychiatry and Psychotherapy, School of Medicine, Technical University of Munich, Munich, Germany; 3 kbo-Inn-Salzach-Klinikum, Wasserburg, Germany; 4 40695 Schön Clinic Bad Aibling Harthausen, Alzheimer Therapy Centre, Bad Aibling, Germany; 5 NorthWestern Mental Health, 90134 Royal Melbourne Hospital, Melbourne, Australia

**Keywords:** Young onset dementia, carers, communication technology, online counselling, Alzheimer's disease, frontotemporal dementia

## Abstract

**Objective:**

Compared to late life dementia, Young Onset Dementia (YOD) has its own distinct challenges, including a lack of specialised and age-appropriate support services. Carers of people with YOD experience higher levels of psychological and physical symptoms, and lower quality of life. This study (RHAPSODY-Plus) assessed the acceptability and feasibility of combining RHAPSODY (Research to Assess Policies and Strategies for Dementia in the Young; a web-based information and skill-building programme for carers of people with YOD) with individually tailored support sessions with health professionals (a social worker and a clinical psychologist) provided via online videoconferencing.

**Methods:**

Participants (n = 20) were informal carers aged over 18 years, who were caring for a person with YOD (either Alzheimer's disease or frontotemporal dementia type). Participants used the RHAPSODY programme for 4 weeks, then attended 2 support sessions. Participants and the health professionals then attended individual feedback sessions. Feedback was collected via open-ended and Likert-style questions.

**Results:**

The majority of carers rated the RHAPSODY-Plus programme as good to very good, demonstrating a high level of acceptability. Positive feedback about the programme included being able to receive personal advice additionally to the information provided in RHAPSODY. The healthcare professionals also thought the programme was acceptable and beneficial for access to support. Some limitations in the feasibility of videoconferencing included network and technical issues and the loss of non-verbal communication.

**Conclusions:**

This online pilot study had a high level of acceptability, demonstrating the potential of an individualised multi-modal intervention for carers of people with YOD which offers opportunities to overcome geographical and service access barriers.

## Introduction

Dementia is an overarching term used to describe a range of diseases that reflect neurodegeneration characterised by a progressive loss of cognitive function.^1^ While dementia typically occurs in later life, some individuals develop the illness in middle age. The onset of dementia symptoms prior to age 65 years is termed Young Onset Dementia (YOD).^2–4^ Alzheimer's disease (AD), vascular dementia and frontotemporal dementia (FTD) are the most common types of dementia diagnosed in people with YOD.^5^

When compared to late-life dementia, YOD presents its own distinct challenges, which necessitate tailored interventions for carers of people with YOD specifically. For example, correct and timely diagnosis may be difficult due to the heterogenous presentation of YOD and presence of complex neuropsychiatric symptoms.^3,6–9^ As YOD often develops during working age, families of people with YOD may face employment and financial challenges, such as loss of household income if the person with YOD has to discontinue their employment, and/or if spouses are required to reduce their working hours or give up work to become a primary carer for the person with YOD.^10–14^ Families of people with YOD may also face emotional and psychological difficulties such as grief and a decline in social connectedness.^15–18^ Further challenges include a lack of specialised and age-appropriate services for this group of patients.^19–21^ Lastly, people with YOD and their families and carers also experience more practical concerns around transportation, socialisation, and family support, particularly for those with younger children.^22^

It is well established that caring for people with dementia can negatively impact the carer's physical and mental health.^23–27^ Evidence suggests that, as a consequence of disease onset in an earlier life stage, carers of people with YOD experience higher levels of carer burden, stress, depression, physical complaints, and a lower quality of life.^4,28^ Several reviews have highlighted the benefits (including a reduction in neuropsychiatric symptoms, and improvement in psychosocial factors) for intervention programmes that target carers of people with dementia (not YOD specifically).^29,30^ However, there is a paucity of research examining the efficacy of psychosocial or non-pharmacological interventions for carers of people with YOD.^22,31^ In addition, the practical concerns many carers and families of people with YOD have, as outlined above, mean that access to existing services is limited to those who live in urban areas or who can travel to research centres and hospitals.

Increasing attention is now being given to support interventions using communication technologies.^32^ Interventions utilising communication technologies via the internet, such as online videoconferencing, could provide a promising alternative to face-to-face services for the provision of information, resources, and support to carers of people with YOD. Interventions using communication technologies may also serve as a means of mitigating some of the logistical difficulties associated with providing face-to-face support services for carers of people with YOD. For example, online videoconferencing has been highlighted as a particularly useful form of communication technology for carers of people with dementia, as it improves carers’ access to support and healthcare information, and can facilitate sharing of the care responsibilities, such as decision making.^33–35^ By using communication technology, carers can therefore obtain support and information in any location, and at a time that is convenient for them. This is also helpful for individuals who live in remote or rural locations with a lack of transportation, professional responsibilities, or who are caring for children. Lastly, given the impact of the COVID-19 pandemic globally, there is now a need more than ever for telehealth and the administration of remote interventions as a means of mitigating the impact of communicable diseases on care and service provision.^36^

The RHAPSODY project (Research to Assess Policies and Strategies for Dementia in the Young) aimed to address the lack of information and support services available for carers of individuals with YOD by developing a web-based information and skill-building programme for this specific group of carers.^37,38^ In a pilot study of the RHAPSODY programme (www.ratgeber-junge-demenz.de), which was conducted across three European countries (France, Germany and the United Kingdom), carers for individuals with YOD were invited to access the RHAPSODY study website and review a series of modules with information and advice specific to YOD, including: an explanation of the nature and medical perspectives of YOD, problem solving, how to deal with challenging behaviour and family difficulties, options for further support, and advice for self-care.^37^

In the initial RHAPSODY study, the programme was well received by the family carers.^38,39^ Feedback included the wish to be able to discuss what was learned via RHAPSODY in the context of their individual carer situation (unpublished data). It was therefore decided, in a next step, to combine the original RHAPSODY study protocol with two additional individual support sessions with relevant health professionals (social worker and clinical psychologist) to explore whether this would address the feedback from family carers (RHAPSODY-Plus). Furthermore, it was decided to explore whether these sessions could be conducted via communication technologies, to mitigate some of the difficulties carers have in attending such appointments. The current study therefore sought to answer the research questions: how feasible and acceptable are the support sessions following RHAPSODY intervention use to both carers of people with YOD and health professionals? How feasible and acceptable is delivery of the support sessions in an online format to both carers and health professionals? Acceptability was defined as the participants’ perceived usefulness of the intervention,^38^ and feasibility as their ability to engage with the intervention without difficulties.

## Methods

### Study design

The current study was a single-site, explorative pilot study with a single intervention group, using quantitative and qualitative data. Using a combination of both was chosen as it enabled detailed collection of feedback on feasibility and acceptability in the original RHAPSODY study.^38^ Being a pilot study with a small sample size, it also enabled the addition of more nuance to the subjective feedback.

### Participants

A convenience sample of 20 carers of people with YOD was recruited. Carers were German-speaking informal carers aged 18 years or over, who were caring for a person with YOD who had received a diagnosis of either AD or FTD in the past 12 months. These criteria were consistent with the group that the RHAPSODY programme was designed for.^38^ Carers were required to have access to a home computer and internet connectivity, basic computer literacy, and literacy in German language to participate.

Health professionals who provided the individualised support sessions included one clinical psychologist and one social worker. These were clinical staff at the Centre for Cognitive Disorders at Technical University of Munich (TUM) with substantial experience in working with patients with YOD and their carers.

### Ethics and screening

The study was completed at the TUM, in Germany, and was approved by the research ethics committee of the Medical Faculty of TUM (# 414/17 S).

Clinicians working at the Centre for Cognitive Disorders at TUM identified and approached suitable carer–patient dyads about the study. Carers who expressed interest in participating were then provided with study information by a research assistant and then provided written consent if they wished to participate. An assessment of their ability to access RHAPSODY and use communication technology on their home computer was then completed with the research assistant. Socio-demographic information including age, gender, employment status, health status, education, and residence were collected.

### Study intervention

The study intervention involved carers being encouraged to use the RHAPSODY e-learning programme (see Table 1) for 4 consecutive weeks, followed by 2 individual support sessions with the social worker and clinical psychologist, respectively. Support sessions were conducted using an online videoconferencing programme (meet.lrz.de des Leibnitz-Rechenzentrums). See Table 2 for the intervention procedure. During the initial 4 weeks while using RHAPSODY, an appointment with a research assistant was scheduled to re-check the carer's ability to utilise the communication technology and RHAPSODY programme. Between the two support sessions, the social worker completed, with the consent of the carer, a handover to the clinical psychologist about what was discussed in Support Session 1.

**Table 1.** Modules in the RHAPSODY e-learning programme for carers of people with YOD.

Module number	Component
1	What is younger onset dementia?
2	Medical explanations
3	Common problems and solutions
4	Management of cognitive and behavioural symptoms
5	Adapting to relationship changes
6	Available care and support
7	Looking after yourself

RHAPSODY: Research to Assess Policies and Strategies for Dementia in the Young; YOD: Young Onset Dementia.

**Table 2.** RHAPSODY-Plus study procedure.

Step	Activity	Time
1	Carer uses RHAPSODY e-learning programme	In own time over 4 weeks
2	Technology support session with research assistant	Within 4 weeks above
3	Support session 1 (with social worker)	60 min
4	Social worker and psychologist handover	N/A
5	Support session 2 (with clinical psychologist)	60 min
6	Feedback session with research assistant	20 min

Note: Steps 3 to 6 were completed within 4 weeks after the end of step 1. RHAPSODY: Research to Assess Policies and Strategies for Dementia in the Young.

### Data collection

An interview guide (see Supplemental material) was developed by consensus of the study team based on addressing the feedback from the RHAPSODY study, and included a combination of open-ended and Likert scale questions. Questions aimed to collect feedback regarding the acceptability, feasibility, and subjective benefits and barriers in relation to the support sessions and use of the online videoconferencing programme. Outcome data was collected from carers in a telephone interview following delivery of the support sessions and within 4 weeks of the conclusion of their period using RHAPSODY. Feedback from the healthcare professionals was obtained at their workplace following delivery of all participants’ support sessions. Audio and visual recording was not used to collect feedback data at the direction of the ethics committee. Instead, the research assistant transcribed participant responses by hand whilst conducting the telephone interview.

### Data analysis

Authors AK and NL (bilingual researchers) translated participant responses from German to English and cross-checked all translations to reach consensus. Descriptive statistics were used for demographics and Likert scale responses. Due to the limited sample of health professionals and short answer nature of the open-ended questions to carers, formal qualitative analysis was not conducted. All carer responses to the open-ended questions have been included in the Results section to avoid bias in reporting. Their responses to the open-ended questions have been used to provide more context to their Likert scale responses, and health professionals’ responses compared to them to show both perspectives. Indications of both the carers’ and health professionals’ willingness to use and recommend the RHAPSODY-Plus programme were taken as measures of acceptability. Their perceived ability to use the programme without difficulties was taken as a measure of its feasibility.

## Results

### Participant demographics

A total of 20 carers completed the study. Carers’ ages ranged from 40 to 77 years with a mean age of 57 years. The age of the individuals with dementia ranged from 41 to 68 years, with a mean age of 56 years. The individuals with dementia had exhibited symptoms an average of 2.2 years prior to diagnosis (range of 1 to 5 years). Further characteristics are detailed in Table 3. More carers were female (n = 13; 65%) than male, and most were partners of individuals with dementia (n = 15; 75%) and were employed (n = 16; 80%). A good spread of education level and area of residence (city vs. countryside) was achieved. In addition, 80% of the carers (n = 16) had used support programmes before.

**Table 3.** Participant demographics.

Characteristic	Number	Percentage
Gender		
Female	13	65
Male	7	35
Relationship to person with YOD		
Partner	15	75
Child	3	15
Sibling	2	10
Speak German at home		
Yes	19	95
No	1	5
Employment status		
Full time	9	45
Part time	7	35
Unemployed	1	5
Retired	2	10
Volunteer	1	5
Educational achievement		
University degree	5	25
Apprenticeship	3	15
High school diploma	7	35
Intermediate school diploma	5	25
Area of residence		
City	10	50
Countryside	10	50
Self-rating of health		
Excellent	0	0
Very good	8	40
Good	7	35
Sufficient	5	25
Bad	0	0
Very bad	0	0
Gender of the individual they are caring for		
Female	10	50
Male	10	50
Diagnosis of the person with YOD		
Alzheimer's disease	12	60
Frontotemporal dementia	8	40

YOD: Young Onset Dementia.

#### Feasibility and acceptability of support sessions

Carers were very positive about the support sessions with both the psychologist and social worker: 85% of the carers rated the session with the psychologist as good (n = 9; 45%) or very good (n = 8; 40%), and 95% rated the session with the social worker as good (n = 5; 25%) or very good (n = 14; 70%).

The majority (n = 18; 90%) of carers thought that the two support sessions were useful. Eighty percent (n = 16) of the carers absolutely agreed that they would most likely use the offer if they could get access to sessions like this and 85% (n = 17) would recommend the sessions to other carers. Only 25% of the carers (n = 5) absolutely agreed while 50% (n = 10) did rather agree that the session helped them to better fulfil their role as a carer.

Seventy percent (n = 14) of the carers thought the session with the psychologist was ‘just right’ in length, with 25% (n = 5) believing it was too short and 5% (n = 1) too long. With the social worker, 75% (n = 15; 75%) found the session to be ‘just right’, while 20% (n = 4) found it too short and 5% (n = 1) too long. Less than 50% of the carers thought the sessions with the psychologist (n = 8; 40%) and social worker (n = 9; 45%) were sufficient. Lastly, 90% (n = 18) of the carers would consider paying for these sessions if needed. Suggested costs ranged from €5 to €100, though many commented that these costs should be covered by insurance and were concerned that the cost would impede either their or others’ uptake and affordability. Others would pay on condition – if the support was long term, the fit between the psychologist/social worker and participant were good, sessions were available on short notice, flexibility in session length could be offered, or only due to the costs saved by not needing to travel.

### Useful aspects of the support sessions

In answers to open-ended questions, the main reasons that carers found the support sessions useful were: being able to ask personal questions, having a personal contact to discuss concerns, and that it was good to talk to someone who is knowledgeable and from the clinic.

Carers’ feedback about what they liked most about the conversation with the psychologist focused on two areas. First, the psychologist provided practical recommendations (such as available AD rehabilitation services in the area); and second, they felt able to discuss and ask about personal situations that they could not necessarily speak to family or friends about and receive help with processing their situation and the diagnosis. Two of the carers (10%) felt reassured about their experiences as these were validated by the psychologist and their fears were taken seriously. Aspects of the conversation with the social worker that carers liked most included that the social worker provided concrete recommendations (e.g. daily coping strategies, managing legal issues, and services options) and offered that they could contact the clinic again should they have further questions.

One carer (5%) noted that the support sessions were useful for those who were not as interested in reading (an aspect of the RHAPSODY e-learning programme), and another mentioned that dealing with text on a computer can be difficult for those having a hard time. Another liked feeling that they could bring others involved in the life of the person with YOD to the support conversation. A factor that carers enjoyed was that the conversations were real and not medically oriented.

### Topics discussed in the support sessions

While the two counselling sessions were individually tailored to the carer's needs, the social worker and psychologist reported different topics that discussions centred around in their respective counselling sessions. The social worker reported that their sessions generally focused on work issues, health insurance and pensions, care options, carer support groups, and care for children. The psychologist reported that their sessions generally focused on how to manage dementia and associated symptoms and behaviours, changes of life perspectives, grief, and future planning.

Some carers reported that it was good to talk to someone with professional experience on behaviours and legal matters (n = 3; 15%), while others (n = 6; 30%) felt that they could gain perspective on their situation due to the novel insights provided and learn how to better care for the person with YOD and manage their own self-care.

### Involving the person with dementia

The psychologist also pointed out that the conversation was more challenging if both the carer and the person with dementia were present as they had to adapt their advice to be more sensitive to the presence of the person with dementia. In contrast, the social worker enjoyed when this happened as they were able to facilitate family discussions about practical aspects of care and encourage active participation of the person with dementia.

### Areas for improvement

A few (n = 3; 15%) carers thought the conversations with the psychologist and social worker were not as helpful as they already felt adequately supported and the sessions did not offer any new or more useful information than what they had already known. The social worker reported that it was difficult to provide additional effective or helpful professional advice to carers of care recipients with very advanced YOD such as those in palliative care, the main reason being that these carers were already well experienced in managing their needs and were usually well engaged with support services.

Aspects that carers mentioned could be improved were having more of a structure for those who did not know what they wanted out of the session, having more information for those outside of the area the clinic was located in or telehealth support options, and including advice on how to garner more social support. One carer mentioned that they would also have liked sessions with a medical expert.

The need for flexibility in session lengths and frequency were often mentioned. Some carers (n = 11; 55%) wanted more regular and/or shorter counselling sessions. One carer (5%) wanted a longer session, noting that it was the first time they had opened up and they desired more, while another (n = 1; 5%) felt the hour may not be enough for some to build rapport and trust. Others (n = 7; 35%) mentioned that needs and issues change with time and the course of dementia progression, so ongoing support would be useful. The suggested length between sessions ranged from every 4 weeks to once a year. However, as mentioned above, some already felt they had adequate support and did not need follow-up, and one would have preferred a combined social work and psychology session.

Both the social worker and psychologist thought that 60 min was enough time to make suggestions to carers and motivate them to get help, but the social worker also suggested that the counselling session should be longer for heavily burdened carers and shorter for knowledgeable carers, and both suggested that more regular counselling sessions should be offered to carers if needed. Both the social worker and psychologist stated that even with such a short period of time, the counselling session was therapeutic and helpful for dealing with personal difficulties. Meanwhile, the psychologist thought that it would be worth exploring the option of small group counselling in order to facilitate mutual exchange between carers.

#### Feasibility and acceptability of use of communication technology

50% (n = 10) of the carers reported having moderate difficulties using the technology. These included poor internet connection, a long wait to boot up programmes, forgotten passwords, empty battery or needing to reboot, and camera issues. One carer (5%) was disappointed that technology issues took up some of their session time. The psychologist commented that some people, especially very old carers, may not be able to handle communication technology well, though a comparison between the carers who reported technology difficulties and those who did not revealed no patterns of differences in age, education level, or location (rural vs. city). A few carers (n = 2; 10%) commented that the technology check was appreciated, as they were new to videoconferencing and may not have been able to access the programme without help, though having it immediately before the support session would have been more useful.

### Beneficial aspects of videoconferencing

Qualitatively, 11 carers (55%) reported that the counselling sessions conducted via videoconferencing worked well. Some carers (n = 4; 20%) explained that the use of this technology suited their situation as they did not need to drive long hours to access counselling services. Several (n = 3; 15%) noted that they were sceptical about videoconferencing at first but ended up having positive experiences.

The social worker was very positive overall about the use of online videoconferencing for the same reason of increasing accessibility to services for carers. The psychologist suggested that online videoconferencing was a valuable option for people with FTD in particular due to the lack of appropriate help available locally for them, a comment echoed by a carer who noted that many areas have little or no FTD support.

### Rapport and engagement in counselling

Both health professionals agreed that being able to see and hear the counsellor had rapport-building advantages over phone counselling and was generally acceptable and beneficial for carers. The social worker explained that carers appeared relaxed, and gradually built up confidence when they communicated with and explained their difficult situation to a person they could see. The social worker explained that seeing the carers’ reactions, emotions, facial expressions, and gestures also enabled them to adjust the method of communication and counselling skills used, though the psychologist expressed some concerns about the loss of non-verbal communication that would otherwise be observed in face-to-face counselling.

The social worker reported that it was easier and the session could get started faster when working with a carer they had known before. When working with carers they did not know, there was an advantage to using videoconference rather than the telephone because it helped the building of a relationship faster, and better addressed the carer's individual issues. The psychologist did not think it was challenging to work with a carer that they had not known before. However, they commented that it would be helpful to have more relevant background information about the client available prior to the conversation, such as their living situation, though the social worker mentioned that a benefit of videoconferencing was being able to see this for themselves. The psychologist noted that the continuity of seeing the same healthcare professional would be important should further sessions be offered.

One carer also mentioned that it was important for them to see the person counselling them, and that a phone session would not have been acceptable to them. A carer also mentioned rapport-building as a reason they would have liked to have just one counsellor who could cover both social work and psychology issues, mentioning that they had to repeat information in the second session. If ongoing support was to be offered, two participants (10%) mentioned preferring to see the same counsellor each time.

## Discussion

To address feedback from carers of people with YOD who completed the RHAPSODY e-learning programme^38,39^ that suggested individualised discussions of what they learned would be helpful (unpublished data), the present study assessed the acceptability and feasibility of two 1h support sessions provided via online videoconferencing following use of RHAPSODY. This pilot study found that in a sample of 20 carers, the majority rated their satisfaction with the RHAPSODY-Plus programme overall as good to very good and found the support sessions helpful; most were willing to recommend the programme to others. In addition, most carers and the two healthcare professionals who provided the support sessions thought the video counselling sessions worked well and were of adequate time to address the carers’ individual concerns. There were some concerns about the loss of non-verbal communicative cues and technology issues. Overall, these findings demonstrate a high level of acceptability and adequate feasibility of the support sessions added to the RHAPSODY programme.

The literature suggests that interventions personalised to carers’ goals and needs are most effective.^40^ It is also reported that active involvement of carers and programmes which utilise a combination of psychological and educational interventions contribute to the best outcomes for carers of people with dementia.^41–44^ Further, the use of multi-modal support in these interventions has been shown to improve overall intervention satisfaction and participation.^41^ Strengths of the RHAPSODY-Plus programme therefore include the combination of both educational and personalised content, and encouragement of the carer to be actively involved through engagement in the counselling sessions. Being able to ask personal questions, receive personal and practical recommendations, having an in-person contact, and having their experiences as carers validated and normalised contributed to the high acceptability ratings of the carers in this study. In addition, provision of technological support and assessment of participant's computer literacy and ability to access the RHAPSODY programme also proved beneficial, consistent with previous research.^33,41^

However, several limitations regarding the feasibility of the RHAPSODY-Plus intervention were identified by the carers and healthcare professionals. Technology issues frequently arose despite carers receiving some technology support from a research assistant (though it could be speculated that more issues could have arisen if this was not provided). This was a surprising finding given the relatively young age of carers in this study. Despite online interventions having some strengths including the ability to provide support to those unable to travel to in-person sessions, these difficulties perhaps highlight that carers of people with dementia may not have the time, financial and/or mental resources to engage in learning about new technologies such as an unfamiliar videoconferencing programme.^45^ Strategies to navigate this could include assessing the carer's current use of technology and increasing the flexibility of the programme to use videoconferencing programmes they are already using (especially after the increase in technology use following the COVID-19 pandemic^46^), and offering some form of in-person support (e.g. small group counselling) in conjunction with videoconferencing for those who would prefer it. The latter was suggested by participants in this study and could have the added benefit of providing carers with the social support of other carers in similar positions.^47^

Another limitation identified by carers was the timing of the intervention, with several carers and the social worker suggesting that the intervention programme may be best suited for carers of newly diagnosed patients. Despite targeting carers of people who had been diagnosed with YOD within the last year, symptom onset varied between 1 and 5 years prior to diagnosis, which means the progression of their family member's YOD may have varied widely between the carers in this study. The literature on support for carers of people with advanced dementia centres on grief and end-of-life support,^48^ and perhaps many of the modules in RHAPSODY and social worker's advice on day-to-day management and services available were not relevant for these carers. It is understood that carers of people with YOD are an underserviced population,^49^ and research about the needs and priorities of this group at different stages both pre-diagnosis and post-diagnosis is still sparse. Further research could lead to education programmes for both different diagnoses and different stages of dementia in future. Tailoring of the type of counselling provided (e.g. more of either social worker or psychologist support, and amount of counselling given) may also assist with further personalising the RHAPSODY-Plus intervention.

A strength of the present study was triangulating the data by collecting feedback from both carers and health professionals regarding the feasibility and acceptability of the support sessions.^50^ This enabled researchers to obtain a more detailed and comprehensive understanding of the acceptability and feasibility of RHAPSODY-Plus. However, several caveats also warrant consideration in interpreting the results of this pilot study. Firstly, the intervention was of a short duration, with one follow-up feedback session, and feedback from participants direct to the research team may have resulted in some desirable responding. Restrictions with the ethical approval of the study also meant that audio recording of the interviews could not be completed, which may have influenced the quality and detail of qualitative responses analysed. Finally, the current study focussed on the acceptability and feasibility of the support sessions, with the knowledge that these sessions were building on the knowledge carers had gained through the RHAPSODY e-learning programme. However, carers did not spontaneously mention the effects of the e-learning programme in response to the interview schedule. For future studies, a focus on the interaction between information gained from the e-learning programme and support sessions would be helpful to better understand how they compliment and build on each other.

## Conclusions

Overall, this pilot study of the RHAPSODY-Plus intervention has shown that the provision of two individualised counselling sessions via online videoconferencing, following use of the RHAPSODY e-learning programme, has a high level of acceptability for carers of people with YOD and the health professionals providing the counselling. The feasibility of the counselling sessions was limited by issues with technology use. Valuable feedback was also provided on improvements that could be made to the programme, including targeting it at carers of people more recently diagnosed with YOD and combining it with face-to-face or group counselling options. Findings from this pilot study can inform future larger trials of online individualised multi-modal interventions for carers of people with YOD.

## Supplemental Material

sj-docx-1-dhj-10.1177_20552076231161962 - Supplemental material for Online counselling for family carers of people with young onset dementia: The RHAPSODY-Plus pilot study Click here for additional data file. Supplemental material, sj-docx-1-dhj-10.1177_20552076231161962 for Online counselling for family carers of people with young onset dementia: The RHAPSODY-Plus pilot study by Stephanie Perin, Rhoda Lai, Janine Diehl-Schmid, Emily You, Alexander Kurz, Maria Tensil, Michael Wenz, Bettina Foertsch and Nicola T Lautenschlager in Digital Health

## References

[1] Rone-Adams S Stern DF Olivier TW, et al. Understanding dementia: etiology, communication, and exercise intervention. Strength Cond J 2013; 35: 88–98.

[2] Koopmans RT Rosness T. Young onset dementia – what does the name imply? Int Psychogeriatr 2014; 26: 1931–1933. 25382199 10.1017/S1041610214001574

[3] Rossor MN Fox NC Mummery CJ, et al. The diagnosis of young-onset dementia. Lancet Neurol 2010; 9: 793–806. 20650401 10.1016/S1474-4422(10)70159-9 PMC2947856

[4] Van Vliet D de Vugt M Bakker C, et al. Impact of early onset dementia on caregivers: a review. Int J Geriatr Psychiatry 2010; 25: 1091–1100. 20957692 10.1002/gps.2439

[5] Vieira RT Caixeta L Machado S, et al. Epidemiology of early-onset dementia: a review of the literature. Clin Pract Epidemiol Ment Health 2013; 9: 88–95. 23878613 10.2174/1745017901309010088 PMC3715758

[6] Armari E Jarmolowicz A Panegyres PK. The needs of patients with early onset dementia. Am J Alzheimers Dis Other Demen 2013; 28: 42–46. 23220922 10.1177/1533317512466690 PMC10697224

[7] Mendez MF. The accurate diagnosis of early-onset dementia. Int J Psychiatry Med 2006; 36: 401–412. 17407994 10.2190/Q6J4-R143-P630-KW41

[8] Tan B Fox S Kruger C, et al. Investigating the healthcare utilisation and other support needs of people with young-onset dementia. Maturitas 2019; 122: 31–34. 30797527 10.1016/j.maturitas.2019.01.003

[9] O’Malley M Carter J Stamou V, et al. Receiving a diagnosis of young onset dementia: a scoping review of lived experiences. Aging Ment Health 2021; 25: 1–12. 31647324 10.1080/13607863.2019.1673699

[10] Chaplin R Davidson I. What are the experiences of people with dementia in employment? Dementia 2016; 15: 147–161. 24419354 10.1177/1471301213519252

[11] Ducharme F Kergoat M Antoine P, et al. The unique experience of spouses in early-onset dementia. Am J Alzheimers Dis Other Demen 2013; 28: 634–641. 23823140 10.1177/1533317513494443 PMC10852552

[12] Evans D. An exploration of the impact of younger-onset dementia on employment. Dementia 2019; 18: 262–281. 27609937 10.1177/1471301216668661

[13] Roach P Drummond N. ‘It’s nice to have something to do’: early-onset dementia and maintaining purposeful activity. J Psychiatr Ment Health Nurs 2014; 21: 889–895. 24841949 10.1111/jpm.12154

[14] Mayrhofer AM Greenwood N Smeeton N, et al. Understanding the financial impact of a diagnosis of young onset dementia on individuals and families in the United Kingdom: results of an online survey. Health Soc Care Community 2021; 29: 664–671. 33745216 10.1111/hsc.13334

[15] Cabote CJ Bramble M McCann D. Family caregivers’ experiences of caring for a relative with younger onset dementia: a qualitative systematic review. J Fam Nurs 2015; 21: 443–468. 25724671 10.1177/1074840715573870

[16] Clemerson G Walsh S Isaac C. Towards living well with young onset dementia: an exploration of coping from the perspective of those diagnosed. Dementia 2014; 13: 451–466. 24339066 10.1177/1471301212474149

[17] Hutchinson K Roberts C Kurrle S, et al. The emotional well-being of young people having a parent with younger onset dementia. Dementia 2016; 15: 609–628. 24784939 10.1177/1471301214532111

[18] Kimura NRS Simões JP Santos RL, et al. Young- and late-onset dementia: a comparative study of quality of life, burden, and depressive symptoms in caregivers. J Geriatr Psychiatry Neurol 2021; 34: 434–444. 32552216 10.1177/0891988720933355

[19] Bakker C De Vugt M Van Vliet D, et al. The use of formal and informal care in early onset dementia: results from the NeedYD study. Am J Geriatr Psychiatry 2013; 21: 37–45. 23290201 10.1016/j.jagp.2012.10.004

[20] Goldberg EL. Filling an unmet need: a support group for early stage/young onset Alzheimer’s disease and related dementias. W V Med J 2011; 107: 64–68. 21702419

[21] Roach P Keady J Bee P. ‘It’s easier just to separate them’: practice constructs in the mental health care and support of younger people with dementia and their families. J Psychiatr Ment Health Nurs 2012; 19: 555–562. 22070795 10.1111/j.1365-2850.2011.01836.x

[22] Richardson A Pedley G Pelone F, et al. Psychosocial interventions for people with young onset dementia and their carers: a systematic review. Int Psychogeriatr 2016; 28: 1441–1454. 27072752 10.1017/S1041610216000132

[23] Gouin JP Glaser R Malarkey WB, et al. Chronic stress, daily stressors, and circulating inflammatory markers. Health Psychol 2012; 31: 264–268. 21928900 10.1037/a0025536 PMC3253267

[24] Vitaliano PP Zhang J Scanlan JM. Is caregiving hazardous to one's physical health? A meta-analysis. Psychol Bull 2003; 129: 946–972. 14599289 10.1037/0033-2909.129.6.946

[25] Wang X Liu S Robinson K, et al. The impact of dementia caregiving on self-care management of caregivers and facilitators: a qualitative study. Psychogeriatrics 2019; 19: 23–31. 30088311 10.1111/psyg.12354

[26] Hvidsten L Engedal K Selbaek G, et al. Quality of life of family carers of persons with young-onset compared to late-onset dementia. Aging Ment Health 2020; 24: 1394–1401. 31106576 10.1080/13607863.2019.1617245

[27] Kishita N Contreras ML West J, et al. Exploring the impact of carer stressors and psychological inflexibility on depression and anxiety in family carers of people with dementia. J Contextual Behav Sci 2020; 17: 119–125.

[28] Rosness T Mjørud M Engedal K. Quality of life and depression in carers of patients with early onset dementia. Aging Ment Health 2011; 15: 299–306. 21271385 10.1080/13607861003713224

[29] Brodaty H Arasaratnam C. Meta-analysis of nonpharmacological interventions for neuropsychiatric symptoms of dementia. Am J Psychiatry 2012; 169: 946–953. 22952073 10.1176/appi.ajp.2012.11101529

[30] Van Mierlo LD Meiland FJM Van der Roest HG, et al. Personalised caregiver support: effectiveness of psychosocial interventions in subgroups of caregivers of people with dementia. Int J Geriatr Psychiatry 2011; 27: 1–14. 21520288 10.1002/gps.2694

[31] Jones B Gage H Bakker C, et al. Availability of information on young onset dementia for patients and carers in six European countries. Patient Educ Couns 2018; 101: 159–165. 28843442 10.1016/j.pec.2017.07.013

[32] Farran CJ Zurawski P Inventor BR, et al. An evidence-based technological caregiver skillbuilding intervention for dementia family caregivers: pilot study. Alzheimers Dement 2017; 13: P836–P837.

[33] Chi N Demiris G. A systematic review of telehealth tools and interventions to support family caregivers. J Telemed 2015; 21: 37–44. 10.1177/1357633X14562734 PMC4486048 25475220

[34] Egan KJ Pinto-Bruno ÁC Bighelli I, et al. Online training and support programs designed to improve mental health and reduce burden among caregivers of people with dementia: a systematic review. J Am Med Dir Assoc 2018; 19: 200–6.e1. 10.1016/j.jamda.2017.10.023 29306605

[35] Etxeberria I Salaberria K Gorostiaga A. Online support for family caregivers of people with dementia: a systematic review and meta-analysis of RCTs and quasi-experimental studies. Aging Ment Health 2020; 25: 1–16. 10.1080/13607863.2020.1758900 32363901

[36] Smith AC Thomas E Snoswell CL, et al. Telehealth for global emergencies: implications for coronavirus disease 2019 (COVID-19). J Telemed 2020; 26: 309–313. 10.1177/1357633X20916567 PMC7140977 32196391

[37] Kurz A Bakker C Böhm M, et al. RHAPSODY – internet-based support for caregivers of people with young onset dementia: program design and methods of a pilot study. Int Psychogeriatr 2016; 28: 2091–2099. 27572272 10.1017/S1041610216001186

[38] Metcalfe A Jones B Mayer J, et al. Online information and support for carers of people with young-onset dementia: a multi-site randomised controlled pilot study. Int J Geriatr Psychiatry 2019; 34: 1455–1464. 31111516 10.1002/gps.5154

[39] Daemen M Bruinsma J Bakker C, et al. A cross-sectional evaluation of the Dutch RHAPSODY program: online information and support for caregivers of persons with young-onset dementia. Internet Interv 2022; 28: 100530. 35433278 10.1016/j.invent.2022.100530 PMC9005959

[40] Tatangelo G McCabe M Macleod A, et al. “I just don’t focus on my needs.” The unmet health needs of partner and offspring caregivers of people with dementia: a qualitative study. Int J Nurs Stud 2018; 77: 8–14. 28982034 10.1016/j.ijnurstu.2017.09.011

[41] Marziali E Garcia LJ. Dementia caregivers’ responses to 2 internet-based intervention programs. Am J Alzheimers Dis Other Demen 2011; 26: 36–43. 21282276 10.1177/1533317510387586 PMC10845422

[42] Schulz R Burgio L Burns R, et al. Resources for Enhancing Alzheimer's Caregiver Health (REACH): overview, site-specific outcomes, and future directions. Gerontologist 2003; 43: 514–520. 12937330 10.1093/geront/43.4.514 PMC2579756

[43] Zarit SH Femia EE. A future for family care and dementia intervention research? Challenges and strategies. Aging Ment Health 2008; 12: 5–13. 18297475 10.1080/13607860701616317

[44] Larochette C Wawrziczny E Papo D, et al. An acceptance, role transition, and couple dynamics-based program for caregivers: a qualitative study of the experience of spouses of persons with young-onset dementia. Dementia 2020; 19: 2714–2731. 31184920 10.1177/1471301219854643

[45] Lorenz K Freddolino PP Comas-Herrera A, et al. Technology-based tools and services for people with dementia and carers: mapping technology onto the dementia care pathway. Dementia 2019; 18: 725–741. 28178858 10.1177/1471301217691617

[46] Ungar R Wu L MacLeod S, et al. The impact of COVID-19 on older adults: results from an annual survey. Geriatr Nurs 2022; 44: 131–136. 35150949 10.1016/j.gerinurse.2022.01.010 PMC8801306

[47] Marziali E Damianakis T Donahue P. Internet-based clinical services: virtual support groups for family caregivers. J Technol Hum Serv 2006; 24: 39–54.

[48] Thompson GN Roger K. Understanding the needs of family caregivers of older adults dying with dementia. Palliat Support Care 2014; 12: 223–231. 23773617 10.1017/S1478951513000461

[49] Carter JE Oyebode JR Koopmans RTCM. Young-onset dementia and the need for specialist care: a national and international perspective. Aging Ment Health 2018; 22: 468–473. 28290708 10.1080/13607863.2016.1257563

[50] Patton MQ. Enhancing the quality and credibility of qualitative analysis. Health Serv Res 1999; 34: 1189–1208. 10591279 PMC1089059

