# Supplementary material for: Online counselling for family carers of people with young onset dementia: The RHAPSODY-Plus pilot study
Source: Digit Health. 2023 Mar 8;9:20552076231161962. doi: 10.1177/20552076231161962 (PMC9998420; doi:10.1177/20552076231161962)
Supplement: sj-docx-1-dhj-10.1177_20552076231161962 - Supplemental material for Online counselling for family carers of people with young onset dementia: The RHAPSODY-Plus pilot study [file sj-docx-1-dhj-10.1177_20552076231161962.docx]

**Supplementary Material: Interview Guides for Feedback Sessions**

**Interview Guide for Healthcare Professionals**

1. Generally, was it a good idea to offer such a project?

2. What did you find difficult?

3. Do you have any suggestions for improvement?

4. How do you rate the reaction of carers to the programme?

5. Which were the carers’ main topics?

6. Were there any materials missing that might have been useful for the counselling session?

7. Was there enough time to make suggestions to the carers and motivate them to get help?

8. Was it possible to deal with personal issues in that short time and via videoconferencing?

9. Was it a problem that the experts did not know the participants previously?

10. What are the differences between counselling via online video conferencing and traditional face-to-face counselling?

11. Do you have additional feedback that was not addressed in the previous questions?

**Interview Guide for Carers**

Open-ended questions

1. What did you think of the program?

2. Were the two face-to-face sessions a useful addition to RHAPSODY?

3. What did you like best about the session with the psychologist or what did you find most helpful?

4. What did you like best about the session with the social worker or what did you find most helpful?

5. What did you like least about the session with the psychologist or what did you find least helpful?

6. What did you like least about the session with the social worker or what did you find least helpful?

7. Do you have any suggestions for improvement?

Likert scale questions

8. How useful did you find the conversation with the psychologist? Very good / good / fair / adequate / inadequate

9. How useful did you find the conversation with the social worker? Very good / good / fair / adequate / inadequate

10. The discussions have helped me to fulfil my role as carer better. Fully agree / rather agree / neither / rather disagree / disagree

11. If I had access to conversations like this I would probably take advantage of them. Fully agree / rather agree / neither / rather disagree / disagree

12. I would recommend these conversations to other carers. Fully agree / rather agree / neither / rather disagree / disagree

13. Duration of the conversation with the psychologist. Way too short / just right / way too long

14. Duration of the conversation with the social worker. Way too short / just right / way too long

Other questions

15. Did you find one conversation with the psychologist was sufficient?

16. Did you find one conversation with a social worker was sufficient?

17. Did you have technical problems?

18. If such conversations were chargeable, would you consider paying for them? If yes, what would be an appropriate price?
